# 

**Published:** 1914-09-26

**Authors:** 


### CONTENTS.

PAGE

**Free Treatment with Free Medicine... ... 685**

**Anti-Typhoid Inoculation in the Army ... 686**

**Hospital and Institutional News ... ... ... 687**

The Transport of Wounded from the Field of Battle to the British Hospital—Welsh War Hospital Appointments—The Last Act of Rheims Cathedral — Unqualified Assistants for Infirmary Staffs—Illness of Mr. Howard Collins—Hospital Appeals in War-time—Hospital Finance in Time of War—A New Cornish Hospital Opened—Donors in Touch with Hospital Boards—New Honorary Secretary of Blackpool Hospital—Unique Gift to the Seamen's Hospital—Nurses' Home or Ward for Wounded?—Lay Hostility to Hospital Efficiency—The Value of Cottage Hospital Mortuaries—Professional Prospects of Medical Volunteers—The Truth About Hospital Trains —The Hospital Class and the Food Question—The Standing Grievance of Southwark Infirmary—The Impotence of the Local Government Board—Dr. Mackinnon Memorial Hospital Opened—Hospital Work in Dundee—The Australian Ambulance Force—Secretary of Worcester Infirmary Retires—The Church Army War Hospital—The Week's Drug Market.

**Tuberculosis Pavilions at Isolation Hospitals 693**

Problems Involved in the Present Arrangements.

[*See over.*]

### CONTENTS—(*continued*).

| | PAGE |
|---|---|
| **Poor-Law Sick Relief in London...** ... ... ... | 694 |
| The Lesson of the Metropolitan Asylums Board Report. | |
| **Hospitals and the War** ... ... ... ... | 695 |
| The Work of London Hospitals and Infirmaries. West End Hospital: News from the Staff in Belgium—A London Secretary as Artillery Officer—New Headquarters of British Red Cross Society—Homerton and Bow Infirmaries. | |
| **Soldiers in the Provincial Hospitals ...** ... | 696 |
| Ashton-under-Lyne: Anti-typhoid Inoculation—Bradford—Bristol: Supplementary Hospital—Cambridge: New Quarters of the 1st Eastern General Hospital—Gravesend and District—Leicester: Further Wounded Arrive—Lincoln: 110 Wounded Received—Sheffield: Embarrassing Sympathisers. | |
| **Good Ambulance Work in Glasgow** ... ... | 698 |
| **The Belgian Refugees at Alexandra Palace** ... | 699 |
| Dr. H. CUFF, Medical Superintendent, on Emergency Administration. | |
| **Hospital Architecture and Construction** ... | 701 |
| Cottage Hospital, Staines. | |
| **Buildings Contemplated** ... ... ... ... | 701 |
| **Round the World** ... ... ... ... ... | 702 |
| Fellow-Workers and the Roll of Honour. | |
| **Public Grants to Schools for Mothers ...** ... | 703 |
| The Board of Education's Proposals. | |
| **Personalia** ... ... ... ... ... ... | 703 |
| **The Beginnings of the London Hospital** ... | 704 |
| A New Light on its Early Days. | |
| **The Institutional Worker** ... .. (Suppl.) | 1 |
| War Matters: Enquiries and Answers. Questions for August and September. Employment and Training. | |
| **The Editor's Letter-Box** ... ... ... ... | 706 |
| The Shortage of Medical Officers: An Appeal to the Colonies—Hospital Appeals in Time of War. | |
| **Institutional Needs** ... ... ... ... ... ... | 706 |
| A Scientific Mattress. | |

